# Radiomics analysis from magnetic resonance imaging in predicting the grade of nonfunctioning pancreatic neuroendocrine tumors: a multicenter study

**DOI:** 10.1007/s00330-023-09957-7

**Published:** 2023-08-08

**Authors:** Hai-Bin Zhu, Hai-Tao Zhu, Liu Jiang, Pei Nie, Juan Hu, Wei Tang, Xiao-Yan Zhang, Xiao-Ting Li, Qian Yao, Ying-Shi Sun

**Affiliations:** 1 Key Laboratory of Carcinogenesis and Translational Research (Ministry of Education/Beijing), Department of Radiology, Peking University Cancer Hospital & Institute, 52 Fu Cheng Road, Hai Dian District, Beijing, 100142 China; 2 Department of Ultrasonography, Peking University First Hospital, Xi Cheng District, 100034 Beijing, China; 3 Department of Radiology, Peking University First Hospital, Xi Cheng District, Beijing, 100034 China; 4 Department of Radiology, Affiliated Hospital of Qingdao University, Shi Nan District, Qingdao, 266000 China; 5 Department of Radiology, First Affiliated Hospital of Kunming Medical University, Wu hua District, Kunming, 650032 China; 6 Department of Radiology, Fudan University Shanghai Cancer Center, Xu Hui District, Shanghai, 200032 China; 7 Department of Oncology, Shanghai Medical College, Fudan University, Xu Hui District, Shanghai, 200032 China; 8 Key Laboratory of Carcinogenesis and Translational Research (Ministry of Education), Department of Pathology, Peking University Cancer Hospital & Institute, Hai Dian District, Beijing, 100142 China

**Keywords:** Pancreatic neoplasms, Neuroendocrine tumors, Radiomics, Diffusion magnetic resonance imaging, Prediction model

## Abstract

**Objectives:**

To explore the potential of radiomics features to predict the histologic grade of nonfunctioning pancreatic neuroendocrine tumor (NF-PNET) patients using non-contrast sequence based on MRI.

**Methods:**

Two hundred twenty-eight patients with NF-PNETs undergoing MRI at 5 centers were retrospectively analyzed. Data from center 1 (*n* = 115) constituted the training cohort, and data from centers 2–5 (*n* = 113) constituted the testing cohort. Radiomics features were extracted from T2-weighted images and the apparent diffusion coefficient. The least absolute shrinkage and selection operator was applied to select the most important features and to develop radiomics signatures. The area under receiver operating characteristic curve (AUC) was performed to assess models.

**Results:**

Tumor boundary, enhancement homogeneity, and vascular invasion were used to construct the radiological model to stratify NF-PNET patients into grade 1 and 2/3 groups, which yielded AUC of 0.884 and 0.684 in the training and testing groups. A radiomics model including 4 features was constructed, with an AUC of 0.941 and 0.871 in the training and testing cohorts. The fusion model combining the radiomics signature and radiological characteristics showed good performance in the training set (AUC = 0.956) and in the testing set (AUC = 0.864), respectively.

**Conclusion:**

The developed model that integrates radiomics features with radiological characteristics could be used as a non-invasive, dependable, and accurate tool for the preoperative prediction of grade in NF-PNETs.

**Clinical relevance statement:**

Our study revealed that the fusion model based on a non-contrast MR sequence can be used to predict the histologic grade before operation. The radiomics model may be a new and effective biological marker in NF-PNETs.

**Key Points:**

- *The diagnostic performance of the radiomics model and fusion model was better than that of the model based on clinical information and radiological features in predicting grade 1 and 2/3 of nonfunctioning pancreatic neuroendocrine tumors (NF-PNETs).*
- *Good performance of the model in the four external testing cohorts indicated that the radiomics model and fusion model for predicting the grades of NF-PNETs were robust and reliable, indicating the two models could be used in the clinical setting and facilitate the surgeons’ decision on risk stratification.*
- *The radiomics features were selected from non-contrast T2-weighted images (T2WI) and diffusion-weighted imaging (DWI) sequence, which means that the administration of contrast agent was not needed in grading the NF-PNETs.*

**Supplementary information:**

The online version contains supplementary material available at 10.1007/s00330-023-09957-7.

## Introduction

Pancreatic neuroendocrine tumors (PNETs) account for 1–3% of pancreatic tumors and rank the second most common malignancies of pancreas [1–3]. Nonfunctioning PNETs (NF-PNETs) are much more common than functioning PNETs, accounting for approximately 70–90% of all PNETs [4]. The World Health Organization (WHO) categorizes PNETs as low (grade 1), intermediate (grade 2), or high grade (grade 3) based on the mitotic rate and Ki-67 index [5]. In general, the risk of tumor progression increases by 2% for every 1% increase in the Ki-67 index [6, 7]. Observation is routinely recommended for grade 1 NF-PNETs, especially those sized < 2 cm [8]. In contrast, grade 2/3 tumors are associated with a poorer prognosis and often require more intensive treatment [9]. It is critical to accurately assess the grade before surgery because the individual therapeutic decision-making has been seen to strongly depend on histologic grade, especially for unresectable tumors. However, it was difficult to ascertain the grade before surgery. To date, endoscopic ultrasonography-guided fine-needle aspiration (FNA) is still the most commonly used strategy to diagnose and grade the tumor, although invasiveness, limited accuracy, and difficulty in reflecting tumor heterogeneity have been reported [10, 11].

MRI has shown great potential as an imaging biomarker to predict the tumor grade of PNETs. For example, the parameter ADC calculated from diffusion-weighted imaging (DWI) was proved to be negatively correlated with tumor grade in the previous study [12]. Recently, histogram analysis of ADC maps was proved to be helpful in predicting PNETs grade, and ADC_entropy_ and ADC_kurtosis_ were the most accurate parameters for identification of high-grade PNETs [13]. In addition, T2-weighted images (T2WI) have been used in the evaluation of many cancers because they can provide more details of anatomical information and the texture features from different scanners were highly reproducible [14, 15]. Kulali et al [16] proved that the low to intermediate signal intensity on T2WI and lower ADC values were significantly correlated with high-grade PNETs because these changes can suggest tumor invasiveness. In addition, T2WI and DWI were the most commonly used non-contrast sequences in clinical which means that the administration of contrast agent was not needed [17].

Radiomics can convert imaging data into high-dimensional quantitative image features using a large number of automatically extracted data-characterization algorithms [18, 19]. Recently, radiomics has been successfully applied for the prediction of tumor grade in PNETs as a noninvasive method. For example, Bian et al [20] demonstrated that the MRI rad-score consisting of seven selected features from arterial and portal venous phase images was significantly associated with the NF-PNET grades, with an area under curve (AUC) of 0.775 and accuracy of 0.701, respectively. However, to the best of our knowledge, there have not been published reports using radiomics analysis based on the most commonly used non-contrast MRI sequences including T2WI and DWI. Thus, the purpose of our research was to assess the value of radiomics features from T2WI and DWI for predicting the grade of NF-PNETs.

## Materials and methods

### Patients

The multicenter retrospective study was derived from 5 hospitals in China. This study was conducted in accordance with the Declaration of Helsinki and was approved by the institutional review board of Peking University Cancer Hospital & Institute (Beijing, China). Informed consent was waived.

**Fig. 1:**
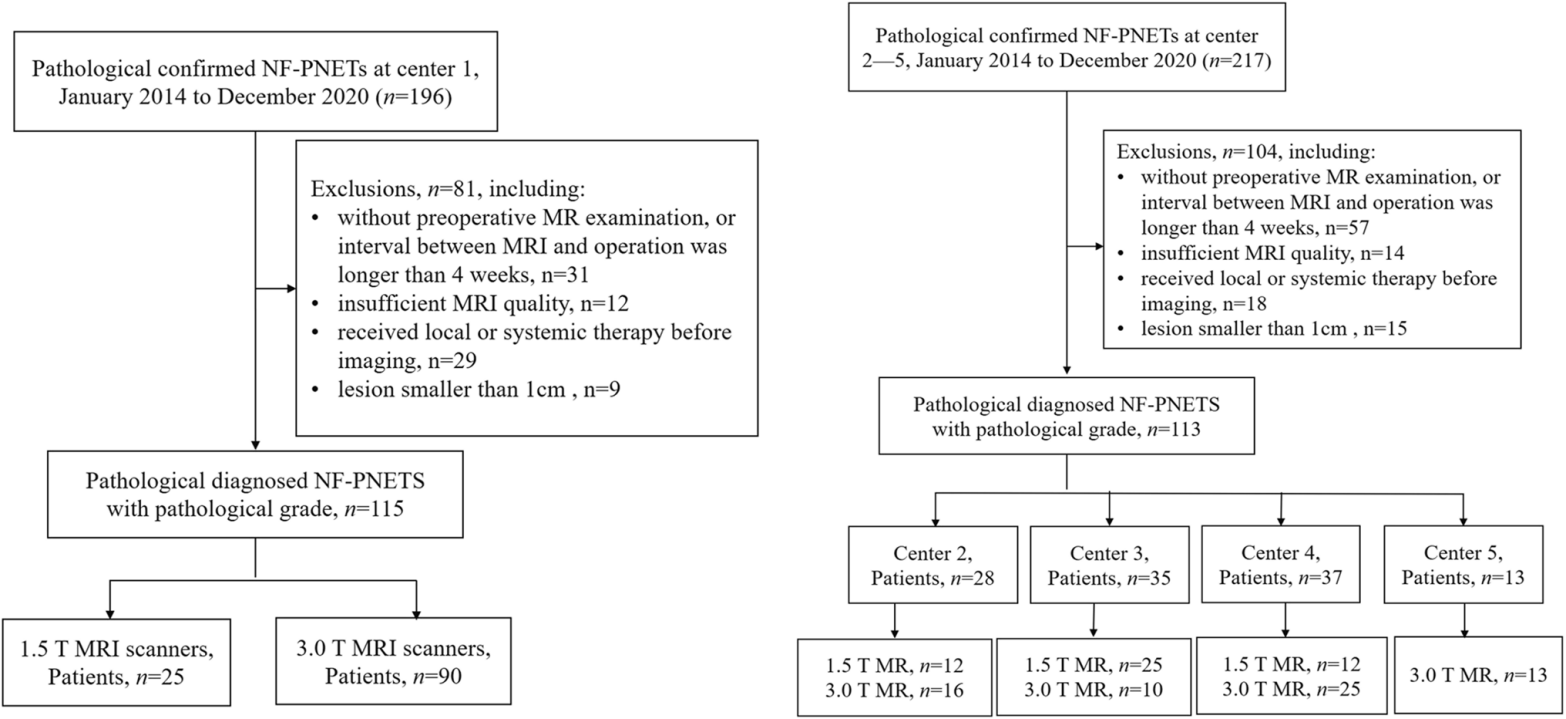
Flowchart of the study of the enrolled patients

The medical records of patients with histologically confirmed NF-PNETs who underwent surgical resection were searched from January 2014 to December 2020 to derive the pathologically confirmed NF-PNETs. Patients were excluded if (1) they had no preoperative MRI or the interval between the MRI examination and operation was longer than 4 weeks; (2) images were not satisfactory for analysis; (3) they received local or systemic therapy before imaging; and (4) the lesion was smaller than 1 cm. The recruitment pathway is shown in Fig. 1.

Baseline clinical information consisting of gender, age, body mass index (BMI), symptom (present or absent), total bilirubin (TB), alanine aminotransferase (ALT), aspartate aminotransferase (AST), fasting blood glucose (FBG), carcinoembryonic antigen (CEA), carbohydrate antigen 199 (CA199), carbohydrate antigen 724 (CA724), and neuron-specific enolase (NSE) were acquired from the medical records. Clinical information was used to construct the clinical model.

### MRI protocols

All examinations were taken on 1.5-T or 3.0-T scanners, using an 8-channel phased array body coil with the patients in the supine position covering the upper abdomen. In the training group, 25 subjects were scanned on a 1.5-T scanner and 90 patients on a 3.0-T scanner. The detail parameters of MRI protocols are listed in Table 1. DWI was performed in all 5 centers with single-shot echo-planar imaging sequence prior to contrast administration with at least a *b* value of 0 and 1000 s/mm^2^.

**Table 1:** MRI scan parameters

	T1-weighted imaging	T2WI-weighted imaging	Diffusion-weighted imaging
Sequences	Axial FSE T1WI	Axial FS-FSE T2WI	Axial SE-EPI
TR/TE (msec)	3.2–6.7/min-9	2200–16,000/64–109	4500–13,333/min-93.3
Flip angle	6–15°	110–142°	90–120°
Thickness/gap (mm)	5–6/0–1	5–6/0–1	5–6/0–1
NEX	1–2	1–4	1–6
Matrix	(224–320) × (192–240)	(288–512) × (256–512)	(96–230) × (128–140)

### MRI feature analysis

The interpretations of MRI, including the qualitative analysis and ROI selection, were done by two radiologists in consensus (H.B.Z. and P.N., both with 12 years’ experience in abdominal MRI). When there was a discrepancy, a senior radiologist (X.Y.Z., with 15 years’ experience in abdominal MRI) was introduced for arbitration, and the result of the arbitration was used in the next analysis. The reviewers were blinded to the clinical information and the pathological reports.

The qualitative features were evaluated first, including (1) tumor location (pancreatic head/neck, body, or tail), (2) signal intensity on T2WI (hypointense, isointense, or hyperintense relative to the surrounding pancreatic parenchyma), (3) maximal axial dimension, (4) tumor margin (regular or irregular) [21], (5) exophytic growth (present or absent), (6) presence of upstream common bile duct dilatation (CBDD, ≥ 10 mm) and/or main pancreatic ductal dilatation (MPDD, ≥ 5 mm) due to tumor compression, (7) presence of hyperenhancement at arterial phase, (8) homogeneity at enhancement, (9) presence of vascular and adjacent organs invasion, and (10) presence of synchronous liver metastases. The 10 qualitative features were analyzed by logistical regression with the forward likelihood ratio (LR) method. The radiological model was constructed in the training group and further validated in the testing cohort.

### Radiomics workflow

#### Image segmentation

The region of interest (ROI) of the whole volume tumor was manually drawn on T2WI and DWI slice by slice with software ITK-SNAP (version 3.8.0, http://www.itksnap.org). Dynamic contrast-enhanced MRI (DCE-MRI) were used as references (if done) for ROI segmentation. Special care was taken to avoid vascular structures, pancreatic duct, and artifacts. ROI was placed on DWI images of the *b* value of 1000 s/mm^2^ and copied to the corresponding ADC maps.

#### Feature extraction

PyRadiomics (Version 3.0.1, https://www.python.org) open-source python package [22] was used for feature extraction. To eliminate the variance among different MRI scanners, image pre-processing was performed using isotropic resample and *Z*-score normalization. A total of 1316 features were extracted from each ROI, including 107 features from the original image and 1209 features from the derived images using filters. Details of the pre-processing steps and the 107 features were described in the supplementary file. Combining features from T2WI and ADC, a total of 2632 features were extracted.

*T* test was used to remove the features that show significant difference (*p* < 0.05) between 1.5- and 3.0-T scanners and the features that show insignificant difference (*p* > 0.05) between grade 1 and grade 2/3 groups. Highly correlated features with the absolute value of Pearson correlation coefficient larger than 0.5 were removed. Logistic regression with least absolute shrinkage and selection operator (LASSO) was used to further remove features. Fivefold cross-validation was performed to determine the hyperparameter in LASSO by maximizing the average accuracy in the training group. More details of feature selection steps were described in the supplementary file. Finally, a radiomics score was obtained by linearly combining the selected features.

The fusion model was constructed from the selected qualitative features in the clinical model and the radiomics score in the radiomics model. Logistical regression was used to calculate the risk of grade 2/3 and visualized as a nomogram. Decision curve of analysis was used to evaluate the net benefit of the model.

### Pathological analysis

Tumor grade was determined by a pathologist (Q.Y., with 13 years of experience) by counting the mitotic rate and Ki-67 index based on the World Health Organization (WHO) 2017 classification [5].

### Statistics

Continuous variables are described as mean values ± standard deviation and were compared with the *t* test. Categorical variables are described as number and percentage and were compared with the Pearson chi-squared test or Fisher’s exact test. Statistical analyses and the logistic regression for the clinical model were performed using SPSS software (version 22.0). Feature selection and the logistic regression for the radiomics model was performed using Python (version 3.6.5). The nomogram for the fusion model, continuous net reclassification index (NRI), and the decision curve of analysis were calculated by R (version 4.1.1) with “rms,” “PredictABEL,” and “rmda” packages. The DeLong test was performed by the MedCalc software (Version 18.2.1). A two-tailed *p* value ≤ 0.05 was considered as statistically significant.

#### Radiomics quality score

Lambin et al developed a 36-point “radiomics quality score” (RQS) metric [23]. The criteria are described in Supplemental Table S1, which shows that the current study had a RQS of 22. In addition, a TRIPOD Checklist following reporting guidelines for prediction model development and validation has also been provided in Supplemental Table S2.

## Results

### Clinical and baseline characteristics

A total of 228 consecutive patients with pathologically proved NF-PNETs were included in this study. One hundred fifteen patients, including 27 grade 1 (23.5%) and 88 grade 2/3 (76.5%) from center 1 were enrolled as the training cohort. The other 113 patients from center 2–5 were enrolled as the testing cohort, including 48 grade 1 (42.5%) and 65 grade 2/3 (57.5%) patients. The patient characteristics are summarized in Table 2. CEA (*p* = 0.010) and NSE (*p* = 0.004) levels were significant differences between the training and testing groups. In addition, baseline MRI characteristics including maximum diameter of the tumor (*p* = 0.048), tumor margin (*p* = 0.005), hyperenhancement at arterial phase (*p* = 0.024), vascular and adjacent tissue involvement (*p* < 0.01), and synchronous liver metastases (*p* < 0.01) were statistically different between the training and testing sets.

**Table 2:** Clinical characteristics, MRI features of patients between different grades of NF-PNETs from the training and testing group

	Training group (*n* = 115)				Validation group (*n* = 113)				*p*^*#*^
G1/G2 + G3	Total	G1 (*n* = 27)	G2 + G3 (*n* = 88)	*p* value	Total	G1 (*n* = 48)	G2 + G3 (*n* = 65)	*p* value	
Gender (male/female)	57/58	11/16	46/42	0.294	54/59	24/24	30/35	0.686	0.788
Age, years	52.55 ± 12.25	52.15 ± 11.98	52.67 ± 12.40	0.847	53.07 ± 14.89	53.56 ± 13.23	52.71 ± 16.09	0.764	0.772
BMI, kg/m^2^	24.13 ± 3.59	24.54 ± 3.59	24.00 ± 3.60	0.500	24.48 ± 3.61	25.15 ± 3.16	23.99 ± 3.87	0.093	0.455
Symptom (present or absent)	66/49	18/9	48/40	0.265	74/36	30/18	44/21	0.566	0.209
NLR	2.89 ± 2.41	2.31 ± 1.03	3.08 ± 2.70	0.162	3.29 ± 4.26	3.18 ± 4.10	3.25 ± 4.18	0.929	0.403
FBG, mmol/L	6.11 ± 2.31	3.72 ± 2.09	6.23 ± 2.37	0.334	5.95 ± 3.80	5.57 ± 3.41	6.26 ± 3.84	0.378	0.726
TB (< 21/≥ 21), μmol/L	84/21	22/4	62/17	0.498	99/14	44/4	55/10	0.261	0.126
ALT (< 40/≥ 40), IU/L	88/17	25/1	63/16	0.065	94/19	43/5	51/14	0.118	0.901
AST (< 40/≥ 40) , IU/L	89/16	25/1	64/15	0.111	104/9	44/4	60/5	0.901	0.092
CEA (< 5/≥ 5), ng/mL	90/11	20/4	70/7	0.298	99/2	40/1	59/1	0.784	0.010*
CA199 (< 37/≥ 37), U/mL	82/20	23/1	59/19	0.029*	93/11	39/3	54/8	0.349	0.070
CA724 (< 5.9/≥ 5.9) , U/mL	72/9	14/2	58/7	0.844	34/6	13/3	21/3	0.588	0.541
NSE (< 16.3/≥ 16.3), ng/mL	56/37	16/3	40/34	0.017*	33/5	16/1	17/4	0.233	0.004*
Tumor location (head or neck/body/tail)	56/30/29	14/7/6	42/23/23	0.906	42/32/39	16/12/20	26/20/19	0.388	0.172
SI on T2WI (hypointense/ isointense/hyperintense)	14/76/25	1/18/8	13/58/17	0.213	5/78/30	4/29/15	1/49/15	0.107	0.094
Maximum diameter of the tumor (mm)	40.84 ± 26.28	31.78 ± 18.61	43.63 ± 27.71	0.040*	33.66 ± 28.34	19.25 ± 10.68	44.31 ± 32.42	0.000*	0.048*
Tumor margin (regular/ irregular)	50/65	22/5	28/60	0.000*	70/43	33/15	37/28	0.201	0.005*
Exophytic growth (present or absent)	69 /46	15/12	54/34	0.590	62/51	18/30	44/21	0.001*	0.433
MPDD or CBDD (present or absent)	37/78	10/17	27/61	0.536	28/85	5/43	23/42	0.002*	0.216
Hyperenhancement at arterial phase (present or absent)	41/74	14/13	27/61	0.045*	57/56	35/13	22/43	0.000*	0.024*
Homogeneity	64/51	22/5	42/46	0.002*	64/49	36/12	28/37	0.001*	0.881
Vascular and adjacent tissue involvement (present or absent)	45/70	3/24	42/46	0.001*	16/97	1/47	15/50	0.002*	0.000*
Synchronous liver metastases (present or absent)	63/52	5/22	58/30	0.000*	10/103	3/45	7/58	0.403	0.000*

*BMI* body mass index, *TB* total bilirubin, *ALT* alanine aminotransferase, *AST* aspartate aminotransferase, *FBG* fasting blood glucose, *NLR* neutrophil-lymphocyte ratio, *CEA* carcinoembryonic antigen, *CA199* carbohydrate antigen 199, *CA724* carbohydrate antigen 724, *NSE* neuron-specific enolase, *MPDD* main pancreatic duct dilatation, *CBDD* common bile duct dilatation

*p < 0.05

^#^Comparisons between training group and validation group

### Clinical model

No statistical independent clinical factor was identified through logistic regression, and the only factor in the model was NSE (OR = 0.035, 95% CI, 0.092–1.333, *p* = 0.124). The AUC of the clinical model in the training group was 0.598 (95% CI, 0.458–0.739) with sensitivity of 63.6% and specificity of 16.7%, which suggested poor diagnostic performance. Further validation in the testing group was not calculated.

### Radiological model

Three radiological characteristics, including tumor boundary, enhancement homogeneity, and vascular invasion, were used to construct the clinical model. The model yielded AUC of 0.884 (95% CI, 0.825–0.942) and 0.684 (95% CI, 0.591–0.778) in the training and testing cohorts, respectively. The sensitivity, specificity, positive predictive value (PPV), negative predictive value (NPV), and accuracy of the model for the training cohort were 62.0%, 100%, 100%, 39.7%, and 0.722, respectively, whereas those of the testing cohort were 32.3%, 95.8%, 91.3%, 51.1%, and 0.714, respectively (Table 3).

**Table 3:** Statistical result of the prediction by the radiological model in the training group from center 1 and testing group from the other 4 centers

	AUC (95% CI)	SEN (95% CI)	SPE (95% CI)	PPV (95% CI)	NPV (95% CI)
Training group (center 1)	0.884 (0.825–0.942)	62.0 (51.2–71.9)	100.0 (85.2–100.0)	100.0 (93.7–100.0)	39.7 (27.0–53.4)
Testing group (center 2–5)	0.684 (0.591–0.778)	32.3 (21.2–45.1)	95.8 (85.7–99.5)	91.3 (72.0–98.9)	51.1 (40.3–61.8)
Center 2	0.577 (0.365–0.788)	21.4 (4.7–50.8)	92.9 (66.1–99.8)	75.0 (19.4–99.4)	54.2 (32.8–74.4)
Center 3	0.717 (0.553–0.880)	80.0 (56.3–94.3)	53.3 (26.6–78.7)	69.6 (47.1–86.8)	66.7 (34.9–90.1)
Center 4	0.671 (0.501–0.842)	30.8 (14.3–51.8)	100.0 (71.5–100.0)	100.0 (63.1–100.0)	37.9 (20.7–57.7)
Center 5	0.963 (0.881–1.000)	80.0 (28.4–99.5)	100.00 (63.1–100.0)	100.0 (39.8–100.0)	88.9 (51.8–99.7)

*CI* confidence interval

### Radiomics model

According to the *T* test result between 1.5- and 3.0-T scanners, 1193 features with significant difference (*p* < 0.05) were removed. Out of the remaining 1439 features, 1047 features were excluded using the *T* test examination and 378 features were excluded using correlation. After feature selection, 14 features were selected. Fivefold cross-validation was used to determine the hyperparameter *α*. The optimal *α* was 0.0231. Ultimately, 4 features were selected in the linear prediction model. The linear expression of the radiomics model is:

$$ \begin{aligned}\mathrm{Radiomics}\mathrm{Score}&=-0.19277293\times \mathrm{ADC}\_\mathrm{original}\_\mathrm{shape}\_\mathrm{Sphericity}-0.01586678\\&\times \mathrm{ADC}\_\mathrm{wavelet}-\mathrm{HHH}\_\mathrm{glcm}\_\mathrm{MCC}+0.09997524\\&\times \mathrm{T}2\mathrm{W}\_\mathrm{gradient}\_\mathrm{firstorder}\_\mathrm{Skewness}-0.06597319\\&\times \mathrm{T}2\mathrm{W}\_\mathrm{logarithm}\_\mathrm{gldm}\_\mathrm{SmallDependenceLowGrayLevelEmphasis}\end{aligned} $$

**Fig. 2:**
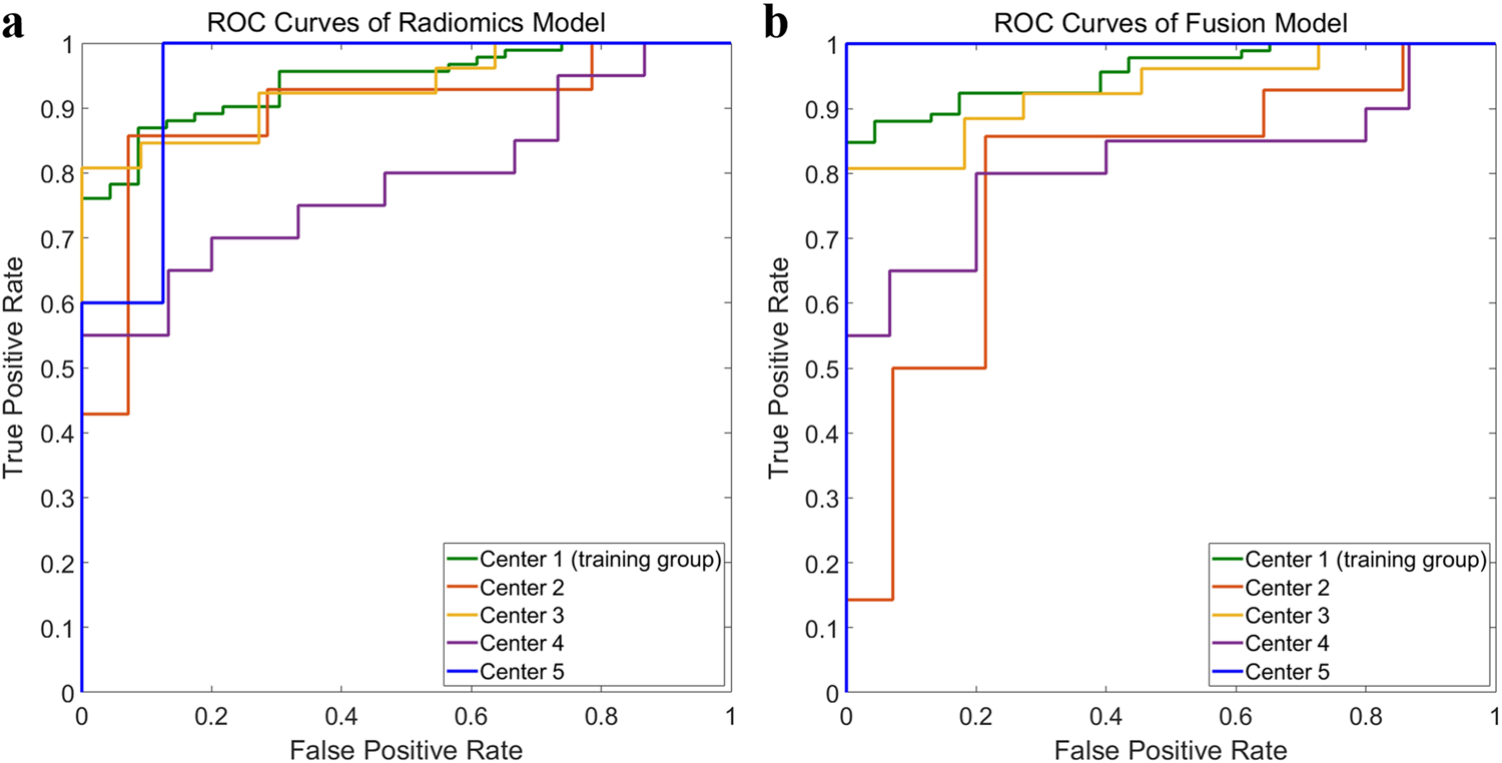
Receiver operating characteristic (ROC) curves for radiomics model (**a**) and fusion model (**b**) in training (center 1) and testing (center 2–5) group

In the training cohort, the radiomics model’s AUC was 0.941 (95% CI, 0.901–0.982), with sensitivity, specificity, PPV, NPV, and accuracy of 87.0%, 91.3%, 97.6%, 63.6%, and 76.9%, respectively. In the testing cohort, the AUC of the model was 0.871 (95% CI, 0.805–0.937), with sensitivity, specificity, PPV, NPV, and accuracy of 84.6%, 81.3%, 85.9%, 79.6%, and 69.2%, respectively (Fig. 2a) (Table 4).

**Table 4:** Statistical result of the prediction by the radiomics model in the training group from center 1 and testing group from the other 4 centers

	AUC (95% CI)	SEN (95% CI)	SPE (95% CI)	PPV (95% CI)	NPV (95% CI)
Training group (center 1)	0.941 (0.901–0.982)	87.0 (78.3–93.1)	91.3 (72.0–98.9)	97.6 (91.5–99.7)	63.6 (45.1–79.6)
Testing group (center 2–5)	0.871 (0.805–0.937)	84.6 (73.5–92.4)	81.3 (67.4–91.1)	85.9 (75.0–93.4)	79.6 (65.7–89.8)
Center 2	0.893 (0.760–1.000)	85.7 (57.2–98.2)	92.9 (66.1–99.8)	92.3 (64.0–99.8)	86.7 (59.5–98.3)
Center 3	0.787 (0.634–0.939)	55.0 (31.5–76.9)	100.00 (78.2–100.0)	100.0 (71.5–100.0)	62.5 (40.6–81.2)
Center 4	0.930 (0.852–1.000)	80.7 (60.6–93.4)	100.0 (71.5–100.0)	100.0 (83.9–100.0)	68.7 (41.3–89.0)
Center 5	0.950 (0.835–1.000)	100.0 (47.8–100.0)	87.5 (47.3–99.7)	83.3 (35.9–99.6)	100.0 (59.0–100.0)

*CI* confidence interval

### Fusion model

**Fig. 3:**
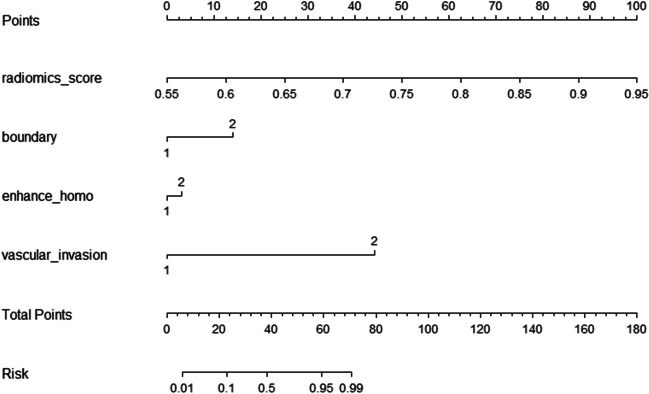
Nomogram of the fusion model that combines radiomics score and 3 qualitative clinical features

**Fig. 4:**
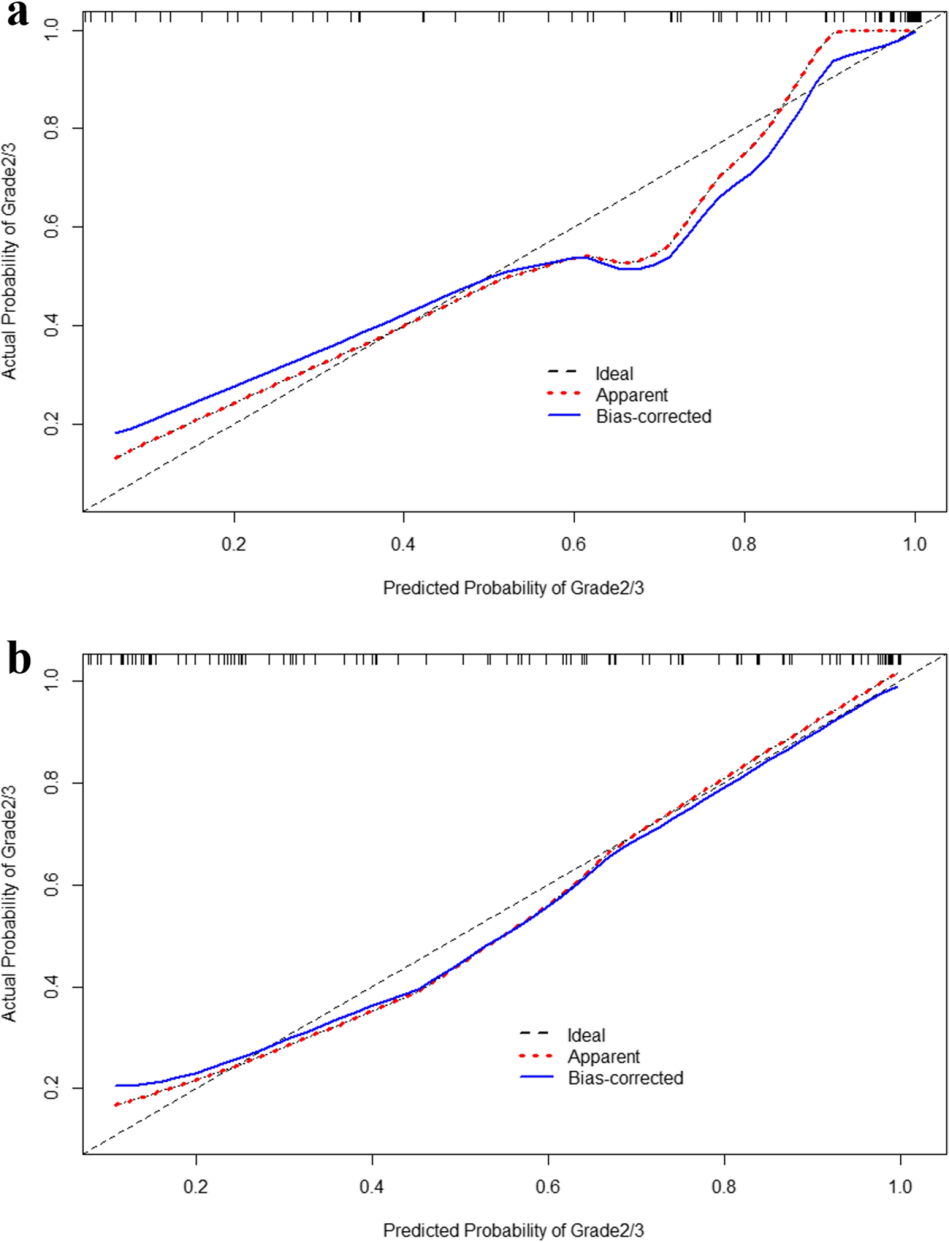
Calibration curves of the fusion model in training group (**a**) and testing group (**b**)

The fusion model visualized in the nomogram (Fig. 3), which combined the radiomics signature and 3 radiological characteristics, yielded the AUC values of 0.956 (95% CI: 0.922–0.989) and 0.864 (95% CI: 0.794–0.935) in the training and testing groups (Fig. 2b) (Table 5). The calibration curves are displayed in Fig. 4. Hosmer-Lemeshow gave a *p* value of 0.991 and 0.582 in the training and testing groups, respectively, indicating good calibration. The fusion radiomics model showed better discrimination than the radiological model (*p* < 0.01). The diagnostic performance of the fusion model was similar to that of the radiomics model, and there were no significant differences between the two models (*p* = 0.521).

**Table 5:** Statistical result of the prediction by the fusion model in the training group from center 1 and testing group from the other 4 centers

	AUC (95% CI)	SEN (95% CI)	SPE (95% CI)	PPV (95% CI)	NPV (95% CI)
Training group (center 1)	0.956 (0.922–0.989)	84.8 (75.8–91.4)	100.0 (85.2–100.0)	100.0 (95.4–100.0)	62.2 (44.8–77.5)
Testing group (center 2–5)	0.864 (0.794–0.935)	87.7 (77.2–94.5)	77.1 (62.7–88.0)	83.8 (72.9–91.6)	82.2 (67.9–92.0)
Center 2	0.791 (0.610–0.972)	85.7 (57.2–98.2)	78.6 (49.2–95.3)	80.0 (51.9–95.7)	84.6 (54.6–98.1)
Center 3	0.817 (0.670–0.964)	80.0 (56.3–94.3)	80.0 (51.9–95.7)	84.2 (60.4–96.6)	75.0 (47.6–92.7)
Center 4	0.930 (0.852–1.000)	80.8 (60.6–93.4)	100.0 (71.5–100.0)	100.0 (83.9–100.0)	68.7 (41.3–89.0)
Center 5	1.000 (1.000–1.000)	100.0 (47.8–100.0)	100.0 (63.1–100.0)	100.0 (47.8–100.0)	100.0 (63.1–100.0)

*CI* confidence interval

### Clinical utility

In the training group, NRI from the radiological model to the radiomics model is 0.696 (95% CI: 0.263–1.128, *p* = 0.002), NRI from the radiological model to the fusion model is 1.370 (95% CI: 1.029–1.711, *p* = 0.000), and NRI from the radiomics model to the fusion model is 1.217 (95% CI: 0.889–1.546, *p* = 0.000). In the validation group, NRI from the radiological model to the radiomics model is 0.878 (95% CI: 0.566–1.190, *p* = 0.000), NRI from the radiological model to the fusion model is 1.073 (95% CI: 0.777–1.370, *p* = 0.000), and NRI from the radiomics model to the fusion model is 0.349 (95% CI: −0.012 to 0.710, *p* = 0.058).

**Fig. 5:**
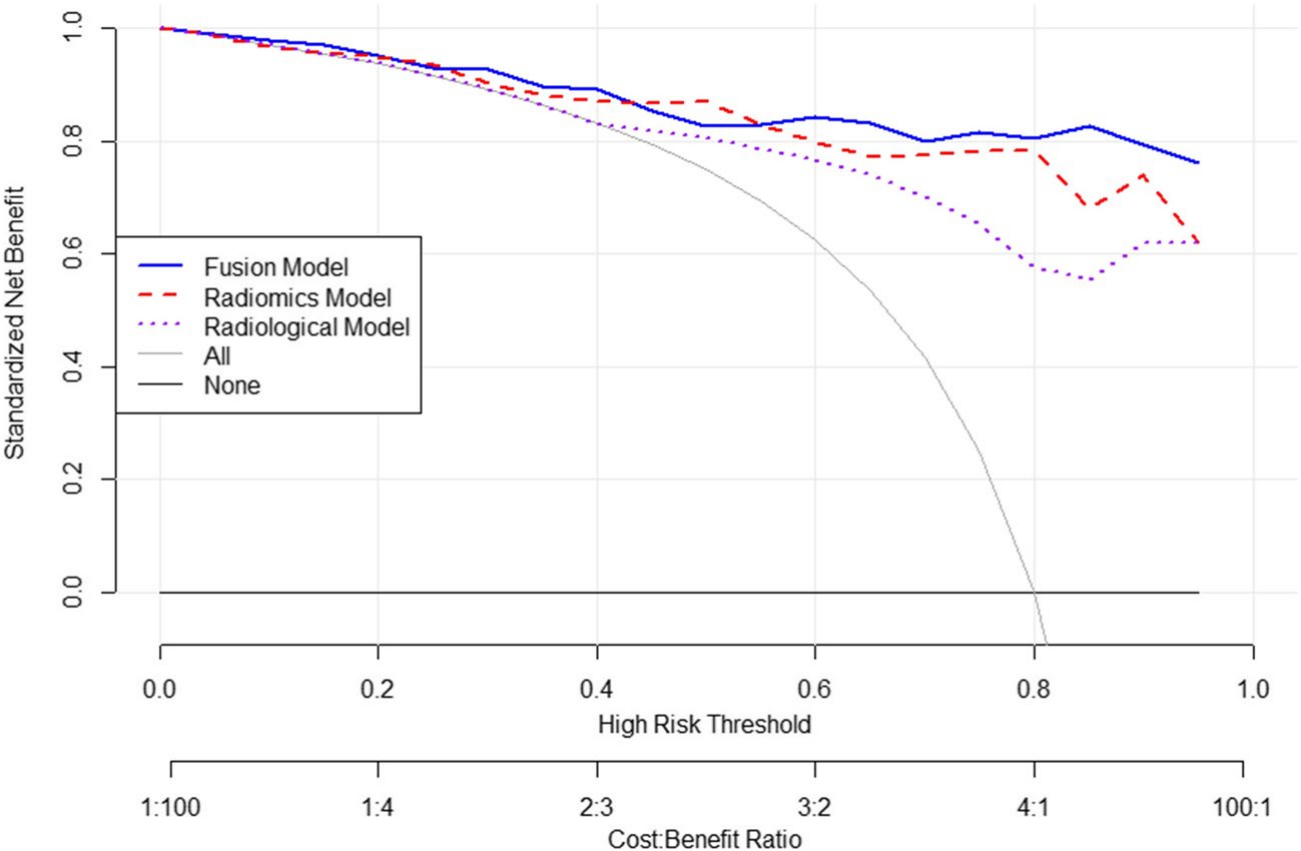
Decision curve of analysis (DCA) for the models. It shows increased standardized net benefit in the whole range of high-risk thresholds

Decision curve of analysis (DCA) is shown in Fig. 5, where the horizontal axis is the risk threshold probability and the vertical direction is the normalized net benefit. The DCA showed that using the fusion model in the current study to distinguish NF-PNET grade is more beneficial than the treat-all-patients scheme or the treat-none scheme in the whole range of threshold. The fusion model performs better than the radiological model in the threshold range of 0.02–1.00. The fusion model performs better than the radiomics model in the threshold range of 0.05–0.20 and 0.27–0.41 and 0.57–1.00.

## Discussion

To predict the grade of NF-PNETs based on clinical information and radiomics from DWI and T2WI, we developed and validated 4 models: clinical model, radiological mode, radiomics model, and fusion model integrated radiological and radiomics model. The diagnostic performance of the radiomics model and the fusion model was better than that of the radiological model in the testing cohort (AUC = 0.871 vs 0.684, *p* = 0.001; AUC = 0.864 vs 0.684, *p* = 0.001). In addition, the fusion model showed similar discrimination in the testing cohort (AUC = 0.864 vs 0.871, *p* = 0.726) compared with the radiomics model. The number of patients correctly classified in the testing cohort (*n* = 113) is 67 for the radiological model, 94 for the radiomics model, and 94 for the fusion model.

Many researchers have investigated the relationship between imaging characteristic and tumor grade. A study conducted by Robertis et al [24] showed ill-defined margin was more common in grade 2/3 tumors with high specificity of 90.3%. Ricci et al [21] showed that the size of tumors and heterogeneous enhancement were related to the risk of grade 2/3 PNETs, indicating grade 1 PNETs showed significantly increased tumor blood flow than higher-grade lesions. Therefore, PNETs with higher grade were more likely to be more aggressive than lower-grade tumors, including ill-defined margin, vascular invasions, and heterogeneous enhancement, which was consistent with our study. However, the results vary a lot and the accuracy remains challenging, as these studies were commonly based on a small-scale study, utilized subjective semi-quantitative imaging parameters, and lacked reliable external validation. Thus, a reliable method that can predict the grade of the tumor preoperatively remains an urgent need.

Radiomics has been widely used in the evaluation of tumor characteristics such as the spatial–temporal heterogeneity [18, 19]. With the quantitative analysis of heterogeneity within tumors, radiomics can help clinicians to assess the intrinsic biologic aggressiveness of tumors and guide individualized treatment. For example, Liang et al [25] constructed a nomogram containing eight radiomics features selected from contrast-enhanced computed tomography (CECT) in combination with clinical stage which showed good performance in the prediction of grade 1 and 2/3 tumors, with AUC of 0.907 and 0.891 in training and testing cohorts, respectively. Similarly, Gu et al [26] found that fusion radiomics model incorporating tumor margin and radiomics signatures was significantly associated with histologic grade, yielding AUC of 0.974 and 0.902 in the training and testing cohorts. However, there were still few studies focused on radiomics analysis on MRI, although multi-parameter MRI exhibited great potential in providing higher soft tissue resolution in comparison with CT. Bian et al [27] selected 14 radiomics features from T2WI and unenhanced T1-weighted fat-suppressed sequences and showed good discrimination between grade 1 and 2/3 tumors in the training (AUC = 0.851) and validation cohort (AUC = 0.736). Recently, Liu et al [28] constructed a model including 6 radiomics features from T2WI and 1 radiomics feature from CECT, which showed better discrimination in the training cohort (AUC = 0.92) and validation cohort (AUC = 0.85) relative to clinical model and the other models using single modality images. Our results were similar with the above results, demonstrating radiomics model were superior to radiological model because it could provide more information and reflect the biological behavior within tumors. In addition, the fusion model could depict more complicated textural information in the tumor heterogeneity, thereby could effectively identify the more aggressive Grade 2/3 NF-PNETs before operation.

In our study, 2632 features were narrowed to only 4 potential predictors to construct the model. One of the significant radiomics predictor is a shape-based feature, namely sphericity. Sphericity has recently been highlighted because it could provide quantitative description of observable shape and its high repeatability [29–31]. Previous studies have shown that sphericity was not only related to the tumor grade, but also can be used as prognostic predictor in many cancers [32–34]. For example, Benedetti et al [32] reported that sphericity was related to high grade, microscopic metastasis, and vascular invasion in PNETs. Other significant radiomics predictor were GLCM features maximal correlation coefficient (MCC) from ADC, gradient first-order skewness and small dependence low gray level emphasis (SDLGE) from T2WI, indicating that the texture complexity of tumor from ADC, the scattered low signals and histogram asymmetry in tumor from T2WI were good predictors of the grade for NF-PNETs. As we all know, higher-grade PNETs tended to be more heterogenous due to increased cystic degeneration, necrosis, and calcification. Therefore, by integrating the radiomics features regarding shape of the whole tumor and heterogeneity, the nomogram achieved good performance in discriminating the grade of PNETs with AUC of 0.941 and 0.871 in the training and testing cohorts, respectively.

Our models have several advantages. First, inter-scanner reproducibility of radiomics features were tested and the most repeatable radiomics features between different scanners were selected. It should be pointed out that reproducibility from different vendors was neglected because previous studies reported that texture features are less sensitive to differences between vendors [35]. Second, the good performance of model in four testing cohort indicated that the model was robust and reliable, further proving the model had a good predictive ability performance in the unfitting new data and could be used in the clinical setting. Thirdly, radiomics features from non-contrast T2WI and DWI were selected and constructed in the model, which means that the administration of contrast agent was not needed, especially beneficial for the patients with chronic kidney insufficient at higher risk to suffer nephrogenic systemic fibrosis (NSF) after administration of gadolinium-based MR contrast agents [36, 37].

This study has several limitations. First, as a retrospective multicenter study, the bias in patient selection and validation is inevitable. Secondly, NF-PNETs confirmed by FNA were excluded because biopsy may lead to misclassification due to intratumoral heterogeneity and sample error. Thirdly, information of other MR sequences was not included in this study although previous studies showed that unenhanced T1-weighted sequence and contrast-enhanced images have great potential for prediction of PNETs grade [25, 28]. DCE-MRI was only used as reference for ROI segmentation in this study. In addition, we did not analyze the relationship between models and survival outcome of the patients. Lastly, manual segmentation of ROI was rather time-consuming. Recently, auto-segmentation of pancreatic tumors in multi-parametric MRI has been introduced which showed comparable performance to expert oncologists using deep convolutional neural networks [38]. Therefore, although the results of our study were promising, more studies are still needed in the future.

In conclusion, we developed a reliable and convenient model integrating radiomics features with radiological characteristics based on non-contrast MRI to predict the grade of NF-PNETs preoperatively from a multicenter study, which can facilitate the surgeon’s clinical decision and guide personalized treatment in NF-PNETs patients.

### Supplementary Information

ESM 1 (PDF 209 kb)

## Abbreviations

ADC: Apparent diffusion coefficient
ALT: Alanine aminotransferase
AST: Aspartate aminotransferase
AUC: Area under curve
BMI: Body mass index
CA199: Carbohydrate antigen 199
CA724: Carbohydrate antigen 724
CEA: Carcinoembryonic antigen
CECT: Contrast-enhanced computed tomography
DCA: Decision curve analysis
DCE-MRI: Dynamic contrast-enhanced MRI
DWI: Diffusion-weighted imaging
FNA: Fine-needle aspiration
ICCs: Intraclass correlation coefficients
LASSO: Least absolute shrinkage and selection operator
MRI: Magnetic resonance imaging
NF-PNETs: Nonfunctioning pancreatic neuroendocrine tumors
NPV: Negative predictive value
NSE: Neuron-specific enolase
NSF: Nephrogenic systemic fibrosis
PACS: Picture archiving and communication system
PNETs: Pancreatic neuroendocrine tumors
PPV: Positive predictive value
ROC: Receiver operating characteristic
ROI: Region of interest
RQS: Radiomics quality score
T2WI: T2-weighted images
WHO: World Health Organization

## Declarations

### Guarantor

The scientific guarantor of this publication is Ying-Shi Sun.

### Statistics and biometry

One of the authors, Xiao-Ting Li, has significant statistical expertise.

### Study subjects or cohorts overlap

No subjects or cohorts have been previously reported.

### Methodology

- Retrospective
- Diagnostic or prognostic study
- Multicenter study
