# Supplementary material for: Radiomics analysis from magnetic resonance imaging in predicting the grade of nonfunctioning pancreatic neuroendocrine tumors: a multicenter study
Source: Eur Radiol. 2023 Aug 8;34(1):90–102. doi: 10.1007/s00330-023-09957-7 (PMC10791720; doi:10.1007/s00330-023-09957-7)
Supplement: Supplementary file 1 — (PDF 209 kb) [file 330_2023_9957_MOESM1_ESM.pdf]

Radiomics analysis from Magnetic Resonance Imaging in prediction the grade of Nonfunctioning pancreatic neuroendocrine tumors: a multicenter study

Electronic Supplementary Material (ESM)

Eur Radiol (2023) Zhu H-B, Zhu H-T, Jiang L, et al.

Image pre-processing

Images were resampled into a resolution of isotropic 1 mm^3 by using the B-spline interpolation. Z-score normalization was performed to T2-weighted images by centering the gray level of all voxels at their mean value with standard deviation, according to the equation $f(x) = (x-\mu_x)/\sigma_x$, where x and f (x) were the original and normalized intensity, respectively. The μ_x and σ_x were the mean and standard deviation of the image intensity values. Normalization was based on all gray values in the image, not just those inside the segmentation. Outliers were removed by setting $x>\mu_x+3\sigma_x$ to $\mu_x+3\sigma_x$ and setting $x<\mu_x-3\sigma_x$ to $\mu_x-3\sigma_x$ respectively.

Feature extraction

The 107 features include 14 shape features, 18 first-order features, 75 texture features (24 gray level co-occurrence matrix features, 14 gray level dependence matrix features, 16 gray level run length matrix features, 16 gray level size zone matrix features, 5 neighboring gray tone difference matrix features). The filters used in this study includes exponential, gradient, logarithm, square, square root, and wavelet filters. The wavelet filters include high-pass and low-pass wavelet filters from each direction, totally 8 types of combination. 18 first-order features and 75 second-order features were extracted from each type of the derived image. For each image modality, 1316 features $(107 + 93 \times 13)$ were extracted. Combining features from T2WI and ADC, totally 2632 features were extracted.

Feature selection

Features were selected by the following 4 steps：

1: Independent samples T-test was performed in the training group to calculate the feature difference between 1.5 T and 3.0 T scanners. The features with $P \geq 0.05$ were considered stable in 1.5 T and 3.0 T scanners and were reserved for further analysis, whereas those with $P < 0.05$ were removed.

2: Independent samples T-test was performed in the training group to calculate the feature difference between G1 and G2/G3 groups. The features with $P \leq 0.05$ were considered significant to discriminate it between the G1 and G2/G3 patients and were reserved for further analysis, whereas those with $P > 0.05$ were removed. The T values of the remaining features were recorded for subsequent analysis.

3: Correlations between each pair of the remaining features were calculated by the Pearson correlation analysis. Any pair of features with the absolute value of Pearson correlation coefficients larger than 0.5 were considered highly correlated. Then the feature with the smaller T value in the t-test was removed.

4: Logistic regression with least absolute shrinkage and selection operator (LASSO) was used to further remove features. LASSO uses a parameter α to control the proportion of L1 regularization and further reduce the number of features. It controls the fraction of L1 regularization. The loss function L(β) of logistic regression with LASSO method is given in Equation (1).

$$L(\beta) = (1-\alpha)\sum_{i=1}^{n}[-y^{(i)}log\left(h_\beta\left(x^{(i)}\right)\right) - (1-y^{(i)})log(1-h_\beta\left(x^{(i)}\right))] + \alpha\|\beta\|_1 \quad (1)$$

β is the weight vector of feature vector x; *n* is the number of subjects; h_β (x) is a sigmoid function defined in Equation (2).

$$h_\beta(x) = \frac{1}{1+e^{-\beta x}} \quad (2)$$

$\|\beta\|_1 = \sum_j^m|\beta_j|$ is L1 regularization that limits the number of included features; *m* is the number of total features; *α* is a number between 0 and 1.

Five-fold cross-validation was used to determine the best *α* in the training group by maximizing the average accuracy. The best *α* was used to train all the subjects in the training group to minimize $L(\beta)$ to obtain the optimal weights $\hat{\beta}$ for the

features x. The radiomics score is defined as $\hat{\beta}x$, the linear combination of the selected features with their optimal weights.

$$radiomics\ score = \hat{\beta}x \qquad (3)$$

Supplementary Table

Supplemental table S1. The criteria and maximal radiomic quality score as well as the actual score of this work

Criteria	Points system	Maximal score	Actual score of this work
Image protocol quality - well-documented image protocols (for example, contrast, slice thickness, energy, etc.) and/or usage of public image protocols allow reproducibility/replicability	+ 1 (if protocols are well-documented)<br>+ 1 (if public protocol is used)	2	1
Multiple segmentations - possible actions are: segmentation by different physicians/algorithms /software, perturbing segmentations by (random) noise, segmentation at different breathing cycles. Analyse feature robustness to segmentation variabilities	1	1	0
Phantom study on all scanners - detect inter-scanner differences and vendor-dependent features. Analyse feature robustness to these sources of variability	1	1	0
Imaging at multiple time points - collect images of individuals at additional time points. Analyse feature robustness to temporal variabilities (for example, organ movement, organ expansion /shrinkage)	1	1	0
Feature reduction or adjustment for multiple testing - decreases the risk of overfitting. Overfitting is inevitable if the number of features exceeds the number of samples. Consider feature robustness when selecting features	− 3 (if neither measure is implemented)<br>+ 3 (if either measure is implemented)	3	3
Multivariable analysis with non radiomics features (for example, EGFR mutation) - is expected to provide a more holistic model. Permits correlating /inferencing between radiomics and non radiomics features	1	1	1
Detect and discuss biological correlates - demonstration of phenotypic differences (possibly associated with underlying gene–protein expression patterns) deepens understanding of radiomics and biology	1	1	1

Cut-off analyses - determine risk groups by either the median, a previously published cut-off or report a continuous risk variable. Reduces the risk of reporting overly optimistic results	1	1	1
Discrimination statistics - report discrimination statistics (for example, C-statistic, ROC curve, AUC) and their statistical significance (for example, p-values, confidence intervals). One can also apply resampling method (for example, bootstrapping, cross-validation)	+1 (if a discrimination statistic and its statistical significance are reported)<br>+1 (if a resampling method technique is also applied)	2	2
Calibration statistics - report calibration statistics (for example, Calibration-in-the-large/slope, calibration plots) and their statistical significance (for example, P-values, confidence intervals). One can also apply resampling method (for example, bootstrapping, cross-validation)	+1 (if a calibration statistic and its statistical significance are reported)<br>+1 (if a resampling method technique is also applied)	2	2
Prospective study registered in a trial database - provides the highest level of evidence supporting the clinical validity and usefulness of the radiomics biomarker	+ 7 (for prospective validation of a radiomics signature in an appropriate trial)	7	0
Validation - the validation is performed without retraining and without adaptation of the cut-off value, provides crucial information with regard to credible clinical performance	- 5 (if validation is missing)<br>+2 (if validation is based on a dataset from the same institute)<br>+3 (if validation is based on a dataset from another institute)<br>+4 (if validation is based on two datasets from two distinct institutes)<br>+4 (if the study validates a previously published signature)<br>+5 (if validation is based on three or more datasets from distinct institutes)	5	5
Comparison to 'gold standard' - assess the extent to which the model agrees with/is superior to the current 'gold standard' method (for example, TNM-staging for survival prediction). This comparison shows the added value of radiomics	2	2	2

Potential clinical utility - report on the current and potential application of the model in a clinical setting (for example, decision curve analysis)	2	2	2
Cost-effectiveness analysis - report on the cost-effectiveness of the clinical application (for example, QALYs generated)	1	1	0
Open science and data - make code and data publicly available. Open science facilitates knowledge transfer and reproducibility of the study	+1 (if scans are open source)<br>+1 (if region of interest segmentations are open source)<br>+1 (if code is open source)<br>+1 (if radiomics features are calculated on a set of representative ROIs and the calculated features and representative ROIs are open source)	4	2
Total score		36	22

Supplemental table S2. TRIPOD Checklist: Prediction Model Development and Validation

Section and Topic	Item #	Checklist item	Location where item is reported
**TITLE**			
Title	1	Identify the study as developing and/or validating a multivariable prediction model, the target population, and the outcome to be predicted	Page 1
**ABSTRACT**			
Abstract	2	Provide a summary of objectives, study design, inclusion patients, radiomics features extraction and models construction, statistical analysis, results, conclusions and highlights	Page 2-6
**INTRODUCTION**			
Background	3	Explain the medical background and rationale for developing and validating the radiomics prediction model, including references to existing models	Pages 7
Objectives	4	Specify the objectives, including whether the study describes the development or validation of the model	Page 8
**Materials and Methods**			
Participants	5	Describe the study design, the inclusion and exclusion criteria of the study	Page 9a

Section and Topic	Item #	Checklist item	Location where item is reported
			Figure 1
Source of data	6	Specify the key elements of the study setting, including the protocols, qualitative features analysis	Page 9b<br>Table 1
Predictors	7	Clearly define all radiomics feathers used in constructing and validating the multivariable prediction model, including how they were measured	Page 10-11
Groups	8	Provide details on how risk groups were created	Page 12a
Statistical analysis methods	9	Specify the methods used to construct the model, compare diagnostic performance between multiple models	Page 12b
**RESULTS**			
Participants	10	Describe the characteristics of the participants (basic demographics, clinical and radiological Characteristics)	Page 13a<br>Table 2<br>Supplementary Table 1

Section and Topic	Item #	Checklist item	Location where item is reported
Model development and performance	11	Present the models to allow predictions for individuals (i.e., all regression coefficients, and model intercept) and report performance measures (with CIs) for the prediction model.	Page 13b-15<br>Table 3-5<br>Figure 2-4
	12	Explain how to the use the prediction model.	Page 16a
**DISCUSSION**			
Discussion	13	Provide a general interpretation of the results in the context of other evidence.<br>Give an overall interpretation of the results, considering objectives, limitations, results from similar studies, and other relevant evidence.	Pages 16b-19a
	14	Discuss any limitations of the study (such as nonrepresentative sample, few events per predictor, missing data).	Page 19b
	15	Discuss the potential clinical use of the model and implications for future research.	Page 20
**OTHER INFORMATION**			
Supplementary information	16	Provide information about the availability of supplementary resources	Supplementary data

Section and Topic	Item #	Checklist item	Location where item is reported
Funding	17	Give the source of funding and the role of the funders for the present study.	Page 21
Competing interests	18	Declare any competing interests of review authors.	Page 21
